# A Dielectric Resonator Antenna with Enhanced Gain and Bandwidth for 5G Applications

**DOI:** 10.3390/s20030675

**Published:** 2020-01-26

**Authors:** Irfan Ali, Mohd Haizal Jamaluddin, Abinash Gaya, Hasliza A. Rahim

**Affiliations:** 1 Wireless Communication Centre, Universiti Teknologi Malaysia, Skudai 81310 UTM, Johor, Malaysia; irfan_lrk_15@yahoo.com (I.A.); abinashgaya@gmail.com (A.G.); 2 Bioelectromagnetic Research Group, School of Computer and Communication Engineering, Universiti Malaysia Perlis, Arau 02600, Perlis, Malaysia; haslizarahim@unimap.edu.my

**Keywords:** dielectric resonator antenna, higher-order mode, quality factor, gain, bandwidth, 5G communication

## Abstract

In this paper, a dielectric resonator antenna (DRA) with high gain and wide impedance bandwidth for fifth-generation (5G) wireless communication applications is proposed. The dielectric resonator antenna is designed to operate at higher-order $TE_{\delta 15}^{x}$ mode to achieve high antenna gain, while a hollow cylinder at the center of the DRA is introduced to improve bandwidth by reducing the quality factor. The DRA is excited by a 50 Ω microstrip line with a narrow aperture slot. The reflection coefficient, antenna gain, and radiation pattern of the proposed DRAs are analyzed using the commercially available full-wave electromagnetic simulation tool CST Microwave Studio (CST MWS). In order to verify the simulation results, the proposed antenna structures were fabricated and experimentally validated. Measured results of the fabricated prototypes show a 10-dB return loss impedance bandwidth of 10.7% (14.3–15.9GHz) and 16.1% (14.1–16.5 GHz) for DRA1 and DRA2, respectively, at the operating frequency of 15 GHz. The results show that the designed antenna structure can be used in the Internet of things (IoT) for device-to-device (D2D) communication in 5G systems.

## 1. Introduction

The presumptions and challenges of the ever-growing traffic explosion drew increased attention toward the significant research activity and development of fifth-generation (5G) wireless communication technology [1]. The most effective way to fulfil the needs of the 5G communication system, which is expected to be launched commercially around 2020 and beyond [2], is to increase bandwidth [3]. Thus, the migration to a higher-frequency band is essential to support the required high data rate on the order of gigabits per second (Gbps) [4]. However, the main problem associated with a higher-frequency band is the high path loss with short distance communication due to the short wavelengths [5]. To overcome these issues, high-gain antennas are required to solve the problems of high path loss and increase the transmission range related to the high-frequency band [6,7]. A microstrip patch antenna (MSA) is considered as a good choice for 5G wireless communication due to its compact size, light weight, low cost, and ease of fabrication [8,9]. However, at higher frequencies, the microstrip patch antenna suffers from low radiation efficiency because of the inherent metallic losses [10,11]. Moreover, it offers low gain and narrow bandwidth. In contrast, dielectric resonator antennas (DRAs) exhibit higher radiation efficiency even at higher frequencies due to the absence of intrinsic conductor loss and surface wave loss [12]. Dielectric resonator antennas, because of their numerous advantages and attractive features like light weight, low cost, and relatively wide impedance bandwidth [13,14,15,16,17], gained increased attention from antenna designers as a good candidate for 5G wireless communication. Additionally, they offer flexible excitation schemes such as coaxial feed probes, microstrip feed lines, aperture coupling, and co-planar waveguides [18,19,20,21].

Several approaches were suggested for the gain enhancement of dielectric resonator antennas in the literature [22,23,24,25,26,27,28]. Multi-segment DRAs or stacked DRAs on top of each other were proposed in References [22,23] to increase the antenna gain. In Reference [23], stacking two rectangular dielectric resonator antennas (rDRAs) with a very high permittivity of 38 and 80 achieved a gain of 6.2 dBi at the operating frequency of 1.5 GHz. However, the major drawback of this approach is that it uses two or more dielectric resonator elements with same or different primitivities; thus, it increases the size of the antenna, as well as the cost. Another technique used for increasing the gain of the DRA is the integration of additional structures [24,25]. In this method, additional structures such as a surface-mounted short horn (SMSH) are placed in the near vicinity of the DR to increase the gain of the antenna. In Reference [25], the gain of a rectangular DRA was enhanced by integrating it with a surface-mounted short horn (SMSH). The major drawback of this approach is the higher complexity, with increased size. Modification of the shape of the dielectric resonator was suggested to enhance the antenna gain [26]. Recently, the higher-order mode technique has been adopted to enhance the gain of DRAs [27,28]. This method has distinct benefits compared to other gain enhancement techniques because it demonstrates high gain and requires a small area with a simple structure, which are attractive features for modern communication systems. However, this approach has the main problem of narrow impedance bandwidth.

In this paper, gain and impedance bandwidth enhancements of DRA are proposed and investigated. Initially, the proposed DRA is designed operating in higher-order $TE_{\delta 15}^{x}$ mode, which enhances the antenna gain. Next, a hollow cylindrical hole is drilled at the center of the DRA to decrease the radiation quality factor (Q-factor), which increases bandwidth. All simulations were performed by using the simulation tool CST Microwave Studio (CST MWS), and the results show good agreement between the simulation and measurement results. To the best of our knowledge, the narrow impedance bandwidth issue of DRAs operating in higher-order mode was not previously addressed in the literature.

The organization of the paper is as follows: Section 2 presents the antenna design and analysis of the proposed DRAs. The measured and simulated results of the antennas are discussed in detail in Section 3. Finally, Section 4 presents the conclusion of the paper.

## 2. Antenna Design and Analysis

**Figure 1:**
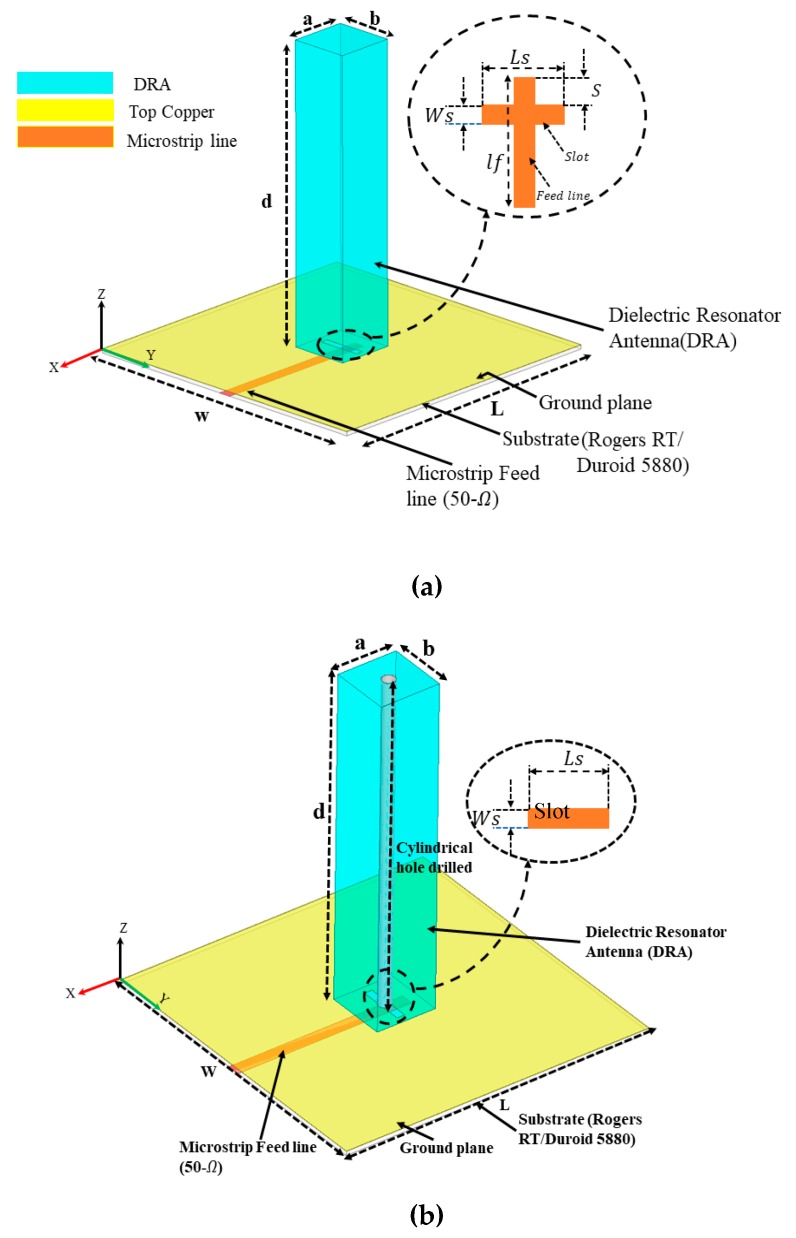
Configuration of the proposed dielectric resonator antennas (DRAs): (**a**) $TE_{\delta 15}$ mode without cylindrical hole (DRA1); (**b**) $TE_{\delta 15}$ mode with a cylindrical hole (DRA2).

The configuration of the proposed DRAs with dimensions of length (a), width (b), and height (d) is shown in Figure 1a,b operating at 15 GHz. The length, width, and height of the designed structure are represented as a×b×d=0.2 λ×0.2 λ×1 λ. The DRA is made of an ECCOS-TOCK HiK material with a dielectric constant ($\varepsilon_{r}$) of 10 and loss tangent (tanδ) of 0.002. The Rogers™ RT/Duroid 5880 substrate with a permittivity of 2.2 and a loss tangent (tanδ) of 0.0009 is used. The thickness of the Rogers substrate is 0.254 mm. Each DRA is mounted on a 20 mm×20 mm=1λ×1λ ground plane and excited by a 50 Ω standard microstrip line with an aperture slot in the ground plane. The ground plane is printed on the top side of the substrate. It is important to mention here that a microstrip feedline is used due to the ease of fabrication. The slot length $l_{s}$, width $w_{s}$ and stub length S are adjusted to match individual antennas. The detailed optimized dimensions of the proposed antenna structures are listed in Table 1. All dimensions are in millimeters (mm). In each case, stub length S was adjusted to optimize the matching impedance of individual DRAs. The resonant frequencies, $f_{o}$, of the ${\mathrm{TE}}_{\delta \mathrm{nm}}^{x}$ mode can be predicted using a dielectric waveguide model (DWM) [29]. The wave numbers $k_{x}$, $k_{y}$, and $k_{z}$ can be deduced by solving the following transcendental equations:

(1) $$k_{x}\tan\left(\frac{k_{x}a}{2}\right)=\sqrt{\left(\varepsilon_{r}-1\right)k_{o}^{2}-k_{x}^{2}},$$

where

(2) $$k_{x}^{2}+k_{y}^{2}+k_{z}^{2}=\varepsilon_{r}k_{o}^{2},$$

(3) $$k_{o}=\frac{2\pi f_{o}}{a},\;k_{y}=\frac{m\pi }{b},\;k_{z}=\frac{n\pi }{d},$$

(4) $$f_{o}=\frac{c}{2\pi \sqrt{\varepsilon_{r}}}\sqrt{k_{x}^{2}+k_{y}^{2}+k_{z}^{2}},$$

where c is the velocity of light, $\varepsilon_{r}$ is the relative permittivity of the DRA, $k_{o}$ is the free space wavenumber, and *m* and *n* are half-wave field variations along the *y*- and *z*-directions, respectively. The symbols $k_{x}$, $k_{y}$, and $k_{z}$ represent the wave numbers in the x-, y-, and z-directions, respectively.

**Figure 2:**
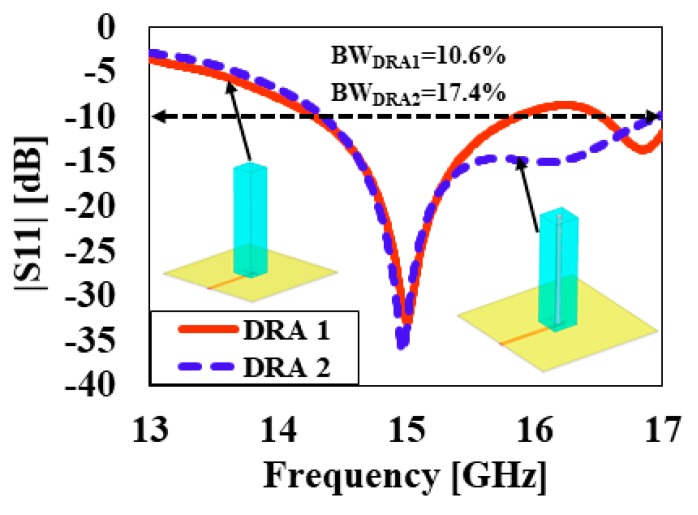
Simulated reflection coefficients |S_11_| of the proposed DRAs.

**Figure 3:**
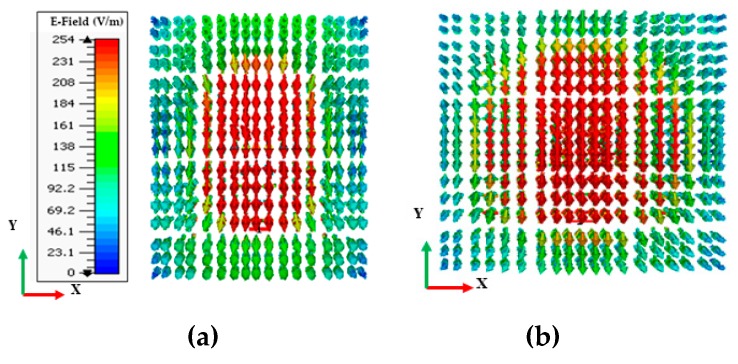
Simulated electric fields (E-fields) of the proposed DRAs at 15 GHz: (**a**) DRA1; (**b**) DRA2.

**Figure 4:**
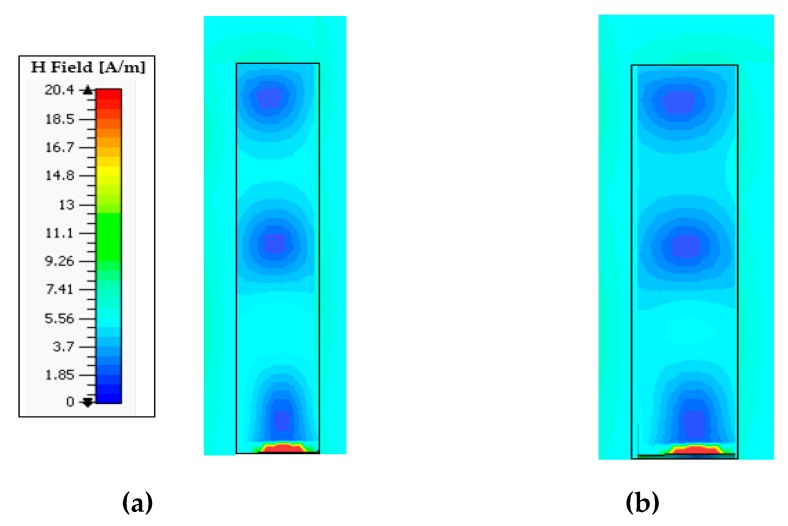
Simulated magnetic fields (H-fields) of the proposed DRAs at 15 GHz: (**a**) DRA1; (**b**) DRA2.

Figure 2 compares the simulated reflection coefficients |S_11_|of the DRA operating in higher-order (${\mathrm{TE}}_{\delta 15}^{x}$) mode without a cylindrical hole (DRA1) and that of the DRA with a cylindrical hole (DRA2). It can be seen from Figure 2 that the DRA operating in higher-order (${\mathrm{TE}}_{\delta 15}^{x}$) mode without a cylindrical hole obtained an impedance bandwidth of 1.6 GHz (10.6%), ranging from 14.3 GHz to 15.9 GHz. The DRA operating in higher-order mode (${\mathrm{TE}}_{\delta 15}^{x}$) with a cylindrical hole at the center achieved a comparatively wider impedance bandwidth of 2.6 GHz (17.4%), operating from 14.3 GHz to 16.9 GHz. The bandwidth of DRA2 was relatively larger than DRA1 because a hollow cylindrical hole was drilled at the center of the DRA2, which reduced the radiation Q-factor of the antenna; therefore, the impedance bandwidth was enhanced. Figure 3a,b present the electric field (E-field) distribution in the *XY* plane for DRA1 (without a cylindrical hole) and DRA2 (with a cylindrical hole), respectively. The electric field distribution was stronger in DRA2 compared to DRA1. The cylindrical hole at the center of DRA2 strengthened the electric field near the center of the DRA. This helped in increasing the bandwidth and efficiency of the DRA. The simulated magnetic fields (H-fields) of both antennas are plotted in Figure 4a,b, respectively, at the operating frequency of 15 GHz. Figure 4a,b show the magnetic field distribution in higher-order $\left(TE_{\delta 15}^{x}\right)$ mode.

## 3. Measurement Results and Discussion

**Figure 5:**
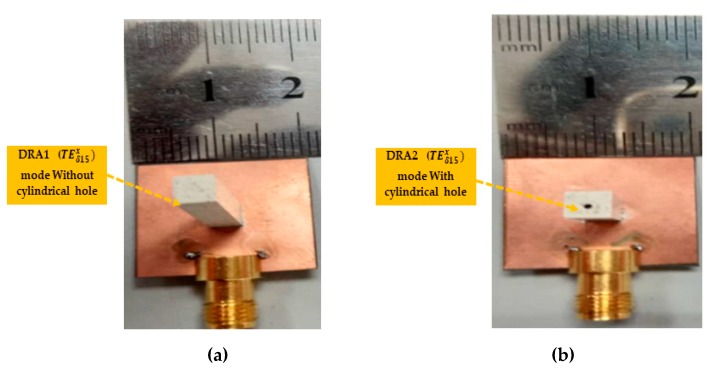

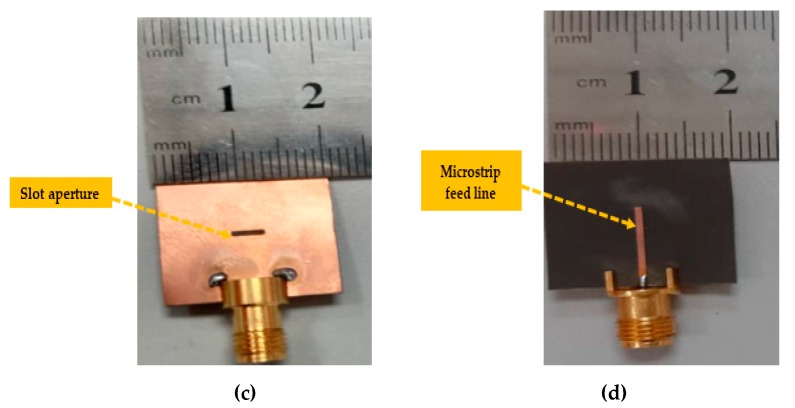
Photos of the fabricated proposed antenna prototypes: (**a**) DRA1 without cylindrical hole (three-dimensional (3D) view); (**b**) DRA2 with cylindrical hole (3D view); (**c**) top view without DRA; (**d**) back view.

**Figure 6:**
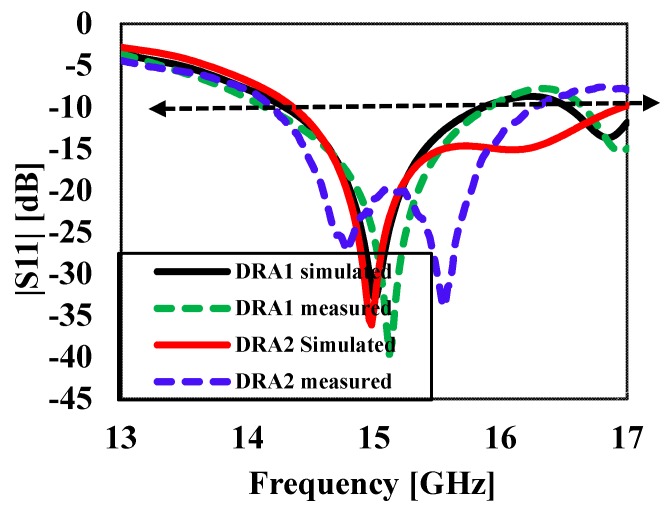
Simulated and measured reflection coefficients |S11| of DRA1 and DRA2.

In this section, the simulated and measured results are analyzed and discussed in detail. The performance of the antenna prototypes was designed and simulated using the commercial three-dimensional (3D) electromagnetic (EM) Computer Simulation Technology (CST) Microwave Studio software. Based on the parameters given in Table 1, prototypes of the proposed DR antennas were fabricated and tested to validate the simulated results. The photographs of the fabricated proposed DR antennas are shown in Figure 5. The reflection coefficients were measured using a vector network analyzer (VNA), while antenna gain and the radiation patterns were measured in an anechoic chamber. The simulated and measured results of reflection coefficients S_11_ of the DRA prototypes are depicted in Figure 6. It can be seen from Figure 6 that DRA1 obtained a simulated and measured −10-dB impedance bandwidth of 10.6% and 10.7%, respectively. On the other hand, DRA2 achieved a simulated and measured −10-dB bandwidth of 17.4% and 16.1%, respectively. The slight difference between the simulated and measured results can be attributed to fabrication imperfections.

For comparison, the measured and simulated results of the reflection coefficient of DRA1 without a cylindrical hole and DRA2 with a cylindrical hole are given in Table 2.

### 3.1. $TE_{\delta 15}^{x}$ without Cylindrical Hole (DRA1)

**Figure 7:**
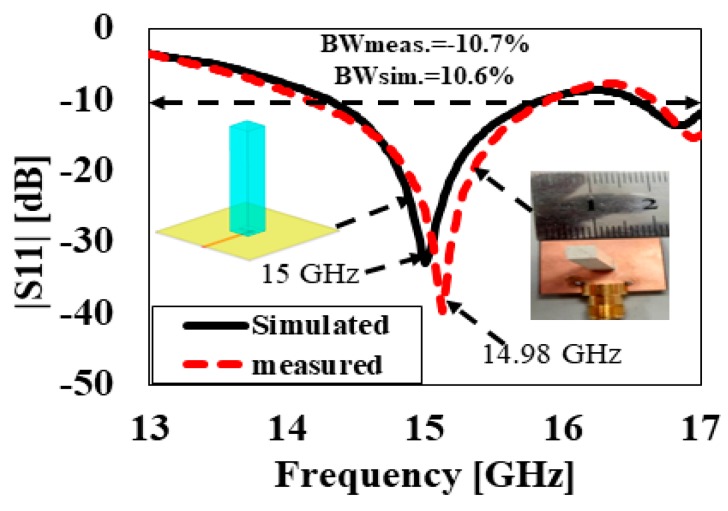
Simulated and measured results of the reflection coefficient |S_11_| of DRA1.

**Figure 8:**
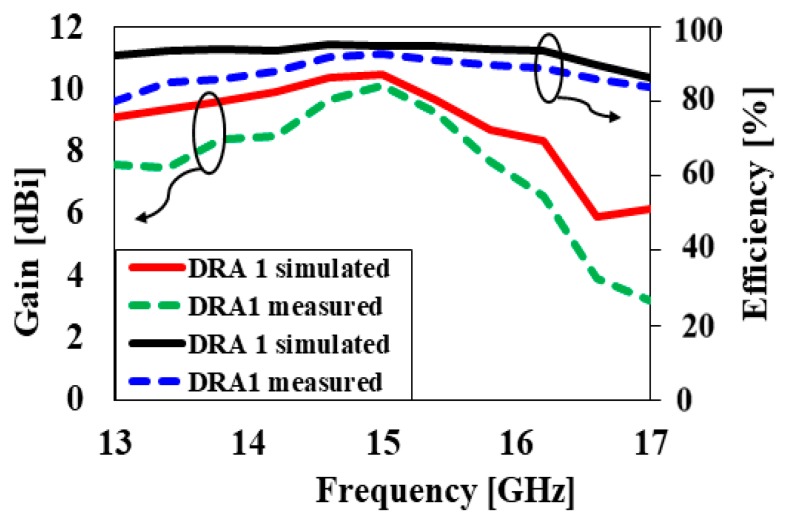
Simulated and measured gain and efficiency versus frequency of DRA1 at 15 GHz.

The simulated and measured reflection coefficients versus frequency plots of the $TE_{\delta 15}^{x}$ mode without a cylindrical hole (DRA1) are represented in Figure 7. It can be seen from Figure 7 that the proposed antenna attained simulated and measured −10-dB impedance bandwidths of 10.6% and 10.7%, respectively. The plot of the simulated and measured antenna gain and radiation efficiency as a function of frequency is depicted in Figure 8. With reference to the plot, the simulated and measured antenna gains were 10.5 dBi and 10.4 dBi, respectively. As presented in Figure 8, the measured and simulated radiation efficiencies were 97% and 95%, respectively. 

**Figure 9:**
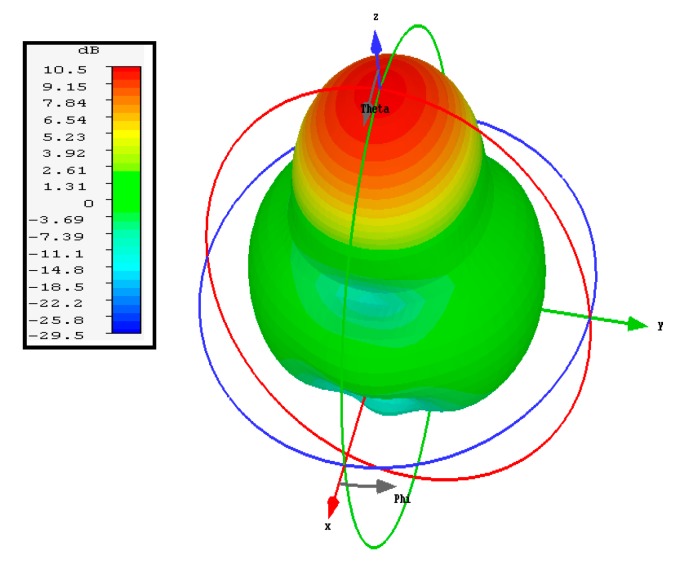
Simulated 3D radiation pattern of DRA1 at 15 GHz.

**Figure 10:**
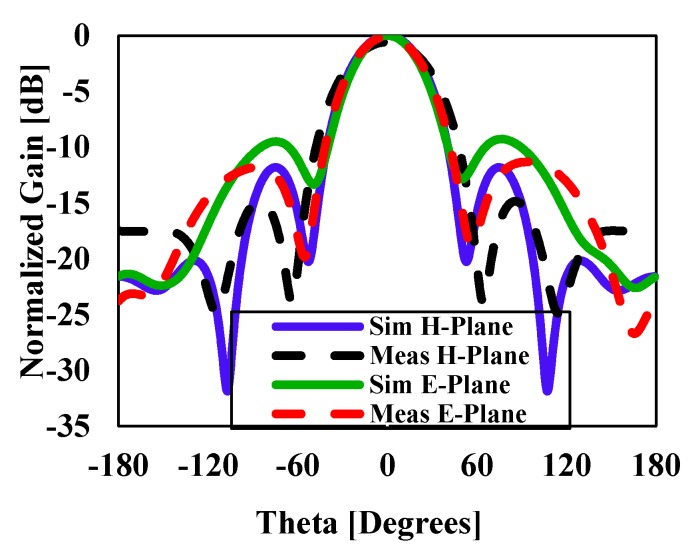
Simulated and measured normalized radiation pattern in the E-plane and H-plane of DRA1 at 15 GHz.

The simulated three-dimensional (3D) radiation pattern of the proposed antenna is illustrated in Figure 9. Figure 10 shows the simulated and measured normalized radiation pattern of the proposed structure in the E- and H-planes at 15 GHz. Figure 10 shows the normalized radiation pattern for DRA1 along the H-plane where the half-power beam width was $49.2^{{}^{\circ}}$ in the major lobe and the radiated power in the side lobe level was −11.8dB. The major lobe is located at $0^{{}^{\circ}}$. In the E-plane, the half-power beam width in the major lobe was $46.4^{{}^{\circ}}$ and the radiated power in the side lobe level was −9.3db.

### 3.2. $TE_{\delta 15}^{x}$ with Cylindrical Hole (DRA2)

**Figure 11:**
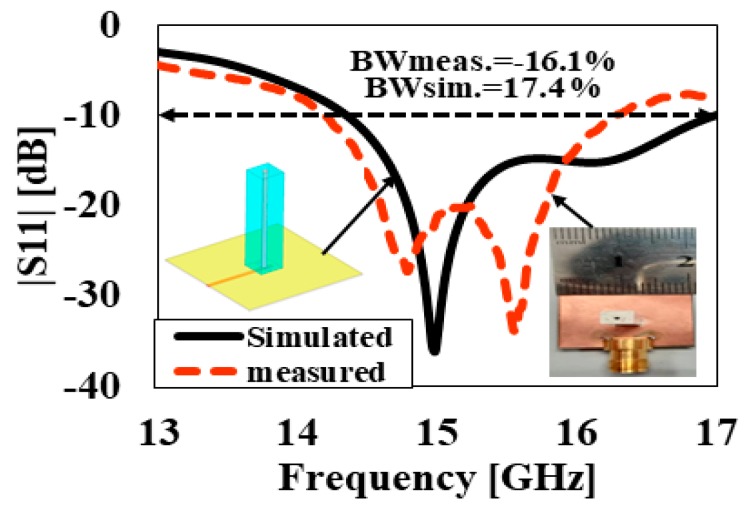
Simulated and measured results of the reflection coefficient |S_11_| of DRA2.

**Figure 12:**
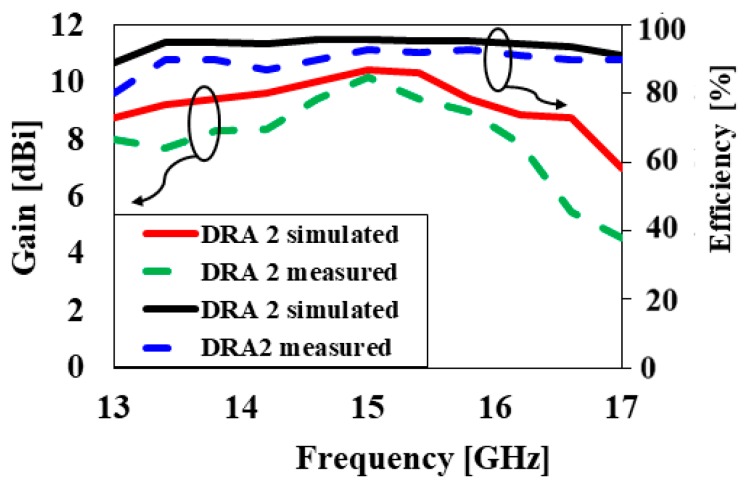
The simulated and measured antenna gain and radiation efficiency versus frequency of DRA2.

**Figure 13:**
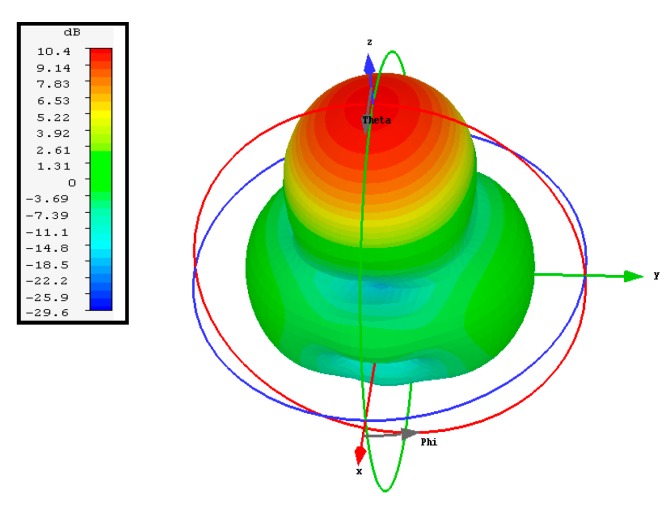
The simulated 3D radiation pattern of the DRA2 at 15 GHz.

**Figure 14:**
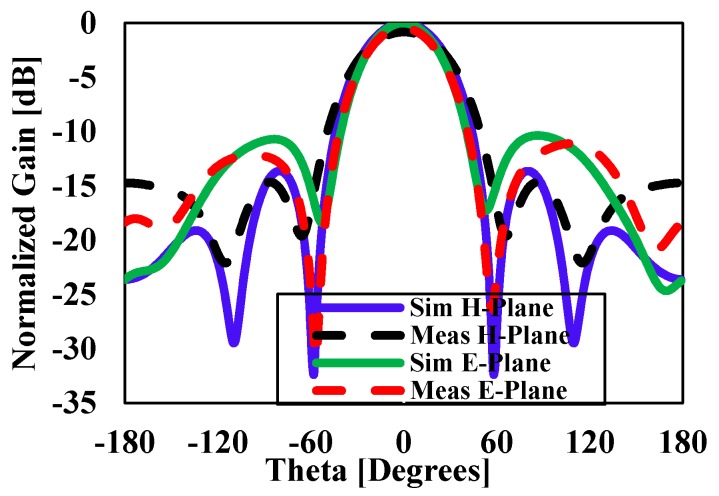
Simulated and measured normalized radiation pattern in the E-plane and H-plane of DRA2 at 15 GHz.

Figure 11 demonstrates the simulated and measured reflection coefficients of the proposed antenna. With reference to Figure 11, the proposed antenna structure achieved simulated and measured bandwidths (S11<−10) of 17.4% (14.3–16.9 GHz) and 16.1% (14.1–16.5GHz), respectively. The slight difference in the measured and simulated results occurred because of the fabrication of the DRA during its assembly process. Figure 12 shows the simulated and measured gain and efficiency of DRA2. It can be seen from Figure 12 that the simulated and measured antenna gains were 10.5 dBi and 10.4 dBi, respectively, while the simulated and measured radiation efficiencies were 98% and 96%, respectively. The simulated three-dimensional (3D) radiation pattern of DRA2 is demonstrated in Figure 13. Figure 14 shows the simulated and measured normalized radiation pattern of DRA2 in the E-plane and H-plane at 15 GHz. Figure 14 shows the normalized radiation pattern for DRA2 with a cylindrical hole along the H-plane, where the half-power beam width was $54.1^{{}^{\circ}}$ in the major lobe, and radiated power in the side lobe level was −13.6 dB. In the E-plane, the half-power beam width in major lobe was $48.6^{{}^{\circ}}$, and the radiated power in the side lobe level was -10.4dB. The major lobe was located at $0^{{}^{\circ}}$.

In Table 3, the simulated and measured results of the proposed DRA prototypes are summarized. An expression that shows the relationship between the bandwidth (BW) and radiation Q-factor (Q) of the DRA is as follows [30]:

(5) $$Q=\frac{\sqrt{VSWR-1}}{VSWR\left(BW\right)}$$

Equation (5) defines the Q-factor in terms of VSWR and bandwidth (BW). With reference to Equation (5), the radiation Q-factor is inversely proportional to the bandwidth. Thus, the equation clearly shows that the radiation Q-factor of the antenna is reduced and, thus, the impedance bandwidth enhanced. In Table 4, the comparison of volume-to-surface ratio of the DRA with and without a cylindrical hole is given.

Finally, a performance comparison between the proposed DRA and previously published work was carried out [31,32,33], as given in Table 5. From Table 5, it can be found that the proposed antenna structure exhibits a wider bandwidth, higher gain, and higher radiation efficiency relative to the aforementioned work. The proposed structure shows better performance compared to previous work.

## 4. Conclusions

A high-gain and wideband dielectric resonator antenna was designed, simulated, fabricated, and experimentally verified. The proposed structure achieved a wide bandwidth and high gain operating in higher-order mode using a new approach of putting a cylindrical hole at the center of the DRA. The DRAs were designed at the operating frequency of 15 GHz. The DRAs were fabricated and measured to validate the proposed design concept. Measured results of the fabricated antenna prototypes showed an impedance bandwidth of 10.7% from 14.3-15.9GHz and 16.1% from 14.1-16.5GHz with a high gain of 10.4dBi for DRA1 and DRA2, respectively. The measured and simulated results of the DRA were in good agreement. Furthermore, the results show that the designed antenna is suitable for future 5G communication applications.

## Figures and Tables

**Table 1:** Optimized dimensions of the proposed DRAs in $TE_{\delta 15}^{x}$ mode with and without a cylinder hole (Unit: mm).

Resonant Modes	a	b	d	$w_{s}$	$l_{s}$	S	Cyl. Hole
DRA1 $TE_{\delta 15}^{x}$ without Cyl. hole	3.84	3.84	19.22	0.34	3.2	1.65	
DRA2 $TE_{\delta 15}^{x}$ with Cyl. hole	3.84	3.84	19.22	0.35	3.3	1.4	0.8

$w_{s}$—width of slot; $l_{s}$—length of slot; S—stub length; Cyl.—cylindrical.

**Table 2:** Comparison of the impedance bandwidth (BW) of the two DRAs in $TE_{\delta 15}^{x}$ mode with and without a cylindrical hole.

Resonant Modes	$f_{r}\left(GHz\right)$	BW (Simulated)	BW (Measured)
DRA1 $TE_{\delta 15}^{x}$ without hole	15	10.6% (14.3–15.9 GHz)	10.7% (14.3–15.9 GHz)
DRA2 $TE_{\delta 15}^{x}$ with cyl. hole	15	17.4%(14.3–16.9 GHz)	16.1%(14.1–16.5 GHz)

$\;f_{r}$—resonant frequency; BW—bandwidth; %—percentage.

**Table 3:** Result summary of simulated and measured results of design antenna structures in $TE_{\delta 15}^{x}$ mode with and without a cylinder hole.

Parameter	Mode	$f_{r}\;\left(GHz\right)$	BW (%)	Gain (dBi)	Efficiency (%)
	DRA1 ($TE_{\delta 15}^{x}$)		10.6%	10.5	97
Simulated		15			
	DRA2 ($TE_{\delta 15}^{x}$) with cyl. hole		17.4%	10.5	98
	DRA1 ($TE_{\delta 15}^{x}$)		10.6%	10.4	95
Measured		15			
	DRA2 ($TE_{\delta 15}^{x}$) with cyl. hole		16.1%	10.4	96

BW—bandwidth; $f_{r}$—resonant frequency.

**Table 4:** Comparison of the volume-to-surface ratio of the two DRAs based on the $TE_{\delta 15}^{x}$ mode with and without a cylinder hole at 15 GHz.

Resonant Mode	Dimensions (a×b×d) (mm)	Cyl. Hole Radius (mm)	$\left(\frac{Volume,V}{Surface,S}\right)\;\mathrm{Ratio}$
DRA1 ($TE_{\delta 15}^{x}$)	3.84×3.84×19.22	-------	0.87
DRA2 ($TE_{\delta 15}^{x}$) with cyl. hole	3.84×3.84×19.22	0.8	0.67

Cyl.—cylindrical; V—volume (mm^3^); S—surface (mm^2^).

**Table 5:** Performance comparisons between the proposed structures and previous work.

Ref	$\varepsilon_{r}$	Shape	Mode	$f_{r}\;\left(GHz\right)$	BW (%)	Gain (dBi)	Eff. (%)	$\mathrm{Area}\;(\lambda^{2})$	Height (λ)
[31]	10	Rect.	$TE_{\delta 15}^{y}$		5.75	5.8	*NM*		0.5λ
			$TE_{\delta 19}^{y}$	24				1.6λ×1.6λ	
					3.4	6.3	*NM*		0.9λ
[32]	11.9	Rect.	$TE_{\delta 17}^{x}$	341	7.3	7.9	74	0.5λ×0.5λ	0.5λ
[33]	10	Rect.	$TE_{\delta 13}^{x}$		7	6.2	46		0.6λ
				135				0.4λ×0.4λ	
			$TE_{\delta 15}^{x}$		7	7.5	42		1λ
PS	10	Rect.	$TE_{\delta 15}^{x}$		10.7	10.4	95		
				15				1λ×1λ	0.9
			$TE_{\delta 15}^{x}$ With cyl. hole		16.1	10.4	96		

$\varepsilon_{r}—$dielectric constant; Rect.—rectangular; $f_{r}$—resonant frequency (GHz); BW—bandwidth (%); gain is measured in dBi; Eff. —efficiency (%); NM—not mentioned; PS—proposed structure.
